# A validated LC-MS/MS analytical method for the quantification of pemigatinib: metabolic stability evaluation in human liver microsomes

**DOI:** 10.1039/d2ra02885a

**Published:** 2022-07-13

**Authors:** Mohamed W. Attwa, Ali S. Abdelhameed, Nawaf A. Alsaif, Adnan A. Kadi, Haitham AlRabiah

**Affiliations:** Department of Pharmaceutical Chemistry, College of Pharmacy, King Saud University Riyadh Saudi Arabia halrabiah@ksu.edu.sa +966 1146 76220 +966 1146 70237

## Abstract

Pemigatinib (PMB) is a small molecule inhibitor of fibroblast growth factor receptor 1 (FGFR1), FGFR2 and FGFR3. On April 17, 2020, the US Food and Drug Administration granted accelerated approval for PMB for the treatment of adults with previously treated, unresectable metastatic or locally advanced cholangiocarcinoma with a fibroblast growth factor receptor 2 (FGFR2) fusion or other rearrangement. PMB is considered the first targeted treatment for cholangiocarcinoma approved in the US. In this study, *in silico* prediction of PMB metabolic stability was done using the WhichP450 module of the StarDrop software package. Further, an LC-MS/MS analytical method was developed for PMB quantification in human liver microsomes (HLM) to experimentally assess metabolic stability. PMB and flavopiridol (FVL), used as an internal standard IS, were resolved using an isocratic mobile phase and a C18 stationary phase. The LC-MS/MS method showed linearity in the range of 5 to 500 ng mL^−1^ in an HLM matrix (*R*^2^ = 0.9995). The lower limit of quantification (LLOQ) was 5 ng mL^−1^, indicating sensitivity. The inter- and intra-day accuracy and precision were within a variability of 10, confirming the reproducibility of the method. The measured *in vitro* half-life and intrinsic clearance of PMB were 27.29 min and 25.40 μL min^−1^ mg^−1^, respectively. PMB showed a moderate extraction ratio suggesting good bioavailability. The developed analytical method is the first LC-MS/MS method specific for PMB quantification with application to metabolic stability assessment.

## Abstract 2

PMB showed a moderate extraction ratio suggesting good bioavailability. The developed analytical method is the first LC-MS/MS method specific for PMB quantification with application to metabolic stability assessment.

## Graphical abstract

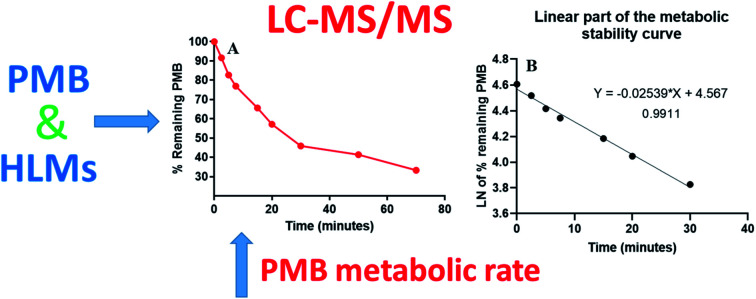

## 1 Introduction

Fibroblast growth factor receptors (FGFRs) are a family of receptor tyrosine kinases with a cytosolic tyrosine kinase domain, an extracellular ligand binding domain, and a transmembrane domain. Binding of fibroblast growth factor (FGF) ligands to the extracellular domain of FGFRs causes dimerization of the receptor, which then activates the intracellular tyrosine kinase domain. This leads to receptor autophosphorylation and activation of multiple downstream signaling pathways including RAS-MAPK, PI3K-AKT, STAT, and PLCγ. FGFR/FGF signaling plays an essential role in cell proliferation, survival, and migration. FGFRs consist of four highly conserved members (FGFR1, FGFR2, FGFR3, and FGFR4) that are expressed in a variety of cells.^1,2^

Genetic alterations such as gene amplification, point mutation or chromosomal translocation/fusion can result in constitutive activation of FGFRs or abnormal ligand-dependent signaling, which can potentially lead to tumor formation. There is strong evidence that dysregulation of FGFRs is involved in multiple tumor types.^3–5^ Due to the predominance of abnormal FGFR activity across a variety of cancer types, FGFR inhibition represents an attractive therapeutic approach for treatment of cancers with genetic FGFR alterations.^6^

Pemigatinib (PEMAZYRE™, Incyte Corporation), a small molecule inhibitor of FGFR1, FGFR2 and FGFR3 with excellent physiochemical properties and pharmacokinetic profile, received accelerated approval in April 2020 in the USA for the treatment of adults with previously treated, unresectable, metastatic or locally advanced cholangiocarcinoma with a FGFR2 fusion or other rearrangement, as detected by a US FDA-approved test.^7^ It is considered the first targeted treatment for cholangiocarcinoma in the USA. Pemigatinib (PMB) received orphan designation for the treatment of myeloid/lymphoid neoplasms with eosinophilia and rearrangement of PDGFRA, PDGFRB or FGFR1, or with PCM1-JAK2 in August 2019 in the USA. PMB is also undergoing clinical development in various countries worldwide for use in several other FGFR alterations (*e.g.* urothelial carcinoma, solid tumors).^8^

Literature search reveals one LC-MS/MS for quantification of PMB with the application in pharmacokinetic study in rat.^9^ The current study showed good accuracy and precision (<10.0%) compared to the published (<13.3%). The mean recovery of PMB the current methodology is 101.7 ± 4.48% that is better compared to the reported method (89.3–92.2%). Also the reported method used gradient elution system for analysis and selective reaction monitoring (SRM: 488.01 → 400.98) for detection which are less selective compared to isocratic elution system and multiple reaction monitoring (MRM: 488 → 401 and 488 → 186) that were used in the current analytical methodology.

The study was conducted to establish LC-MS/MS analytical methodology for the quantification of PMB in human liver microsomal (HLM) matrix similar methods were applied previously in the estimation of the intrinsic clearance (CL_int_) and *in vitro* half-life (*t*_1/2_) of other tyrosin kinase inhibitors.^10^ Such studies can provide more information about the metabolic fate of PMB and to permit prediction of its *in vivo* bioavailability using three models: parallel tube, venous equilibrium, and dispersion models.^11,12^ Here, the PMB *in vitro* CL_int_ and *t*_1/2_ in HLM were computed by an ‘*in vitro t*_1/2_’ approach using the ‘well-stirred’ model^13,14^ If the drug has fast metabolic rate, it will have low *in vivo* bioavailability and a short window of action.^15–18^

## 2 Material and methods

### 2.1 Materials and instruments

Pooled HLM (M0567) from male donors (20 mg mL^−1^ in 250 mM sucrose buffer) were procured from Sigma-Aldrich (St. Louis, MO, USA) and stored at −70 °C until use. All solvents used in the study were of HPLC grade. All chemicals and reference powders were of analytical (AR) grade. Pemigatinib (99.88%) and flavopiridol (99.72%) were procured from MedChem Express Company (Princeton, NJ, USA). Acetonitrile and formic acid were purchased from Sigma-Aldrich (St. Louis, MO, USA). Water (HPLC grade) was obtained from an in-house Milli-Q plus purification equipment that was procured from Millipore (Billerica, MA, USA). Waters Acquity UPLC [serial number H10UPH model code UPH] and Acquity TQD MS [serial number QBB1203 and model code TQD] were used for chromatographic separation and mass detection of analyte peaks. The LC-MS/MS system was controlled by MassLynx 4.1 Data acquisition, processing and reporting were automatically performed using ‘QuanLynx’ included in the MassLynx Software package (Version 4.1, SCN 805). Mass tuning was assisted with IntelliStart®. Rotary pump (SV40B; Murrysville, PA, USA) was used for generating vacuum and a nitrogen generator from Peak Scientific (Renfrewshire, Scotland, UK) was used for supplying desolvation gas. Argon gas of 99.999% purity was obtained locally.

### 2.2 *In silico* metabolic stability evaluation

*In silico* metabolic stability of the substrate was evaluated using the WhichP450 module (StarDrop software package) from Optibrium Ltd. (Cambridge, MA, USA). The outcomes was presented as composite site lability (CSL) value that indicates the degree of metabolic prediction.^19–21^

### 2.3 LC-MS/MS analytical methodology

#### 2.3.1 Liquid chromatography

Liquid chromatography analytical parameters used for separating analytes (PMB and FLV), including mobile phase composition, stationary phase nature and pH were optimized. The pH of 0.1% formic acid solution was 3.2 as ammonium formate (10 mM) with a higher pH (4.0, 4.2, 4.5) caused peak tailing and unnecessarily long running time. The mobile phase consisted of 30% ACN and 70% aqueous solution (0.1% formic acid in water) at a flow rate of 0.3 mL min^−1^. Increasing the percentage of ACN resulted in overlapped peaks and a poor separation, while decreasing ACN resulted in longer running times. Different stationary phases were tested, such as HILIC columns (polar columns), on which, neither PMB nor FLV were retained, and optimal results were achieved using a ZORBAX Eclipse plus-C18 column (i.d. 2.1 mm, particle size 1.8 μm and 50 mm length) at 22 ± 2 °C. Injection volume and run time were 5 μL and 2 min, respectively.

#### 2.3.2 Mass spectrometry

**Fig. 1:**
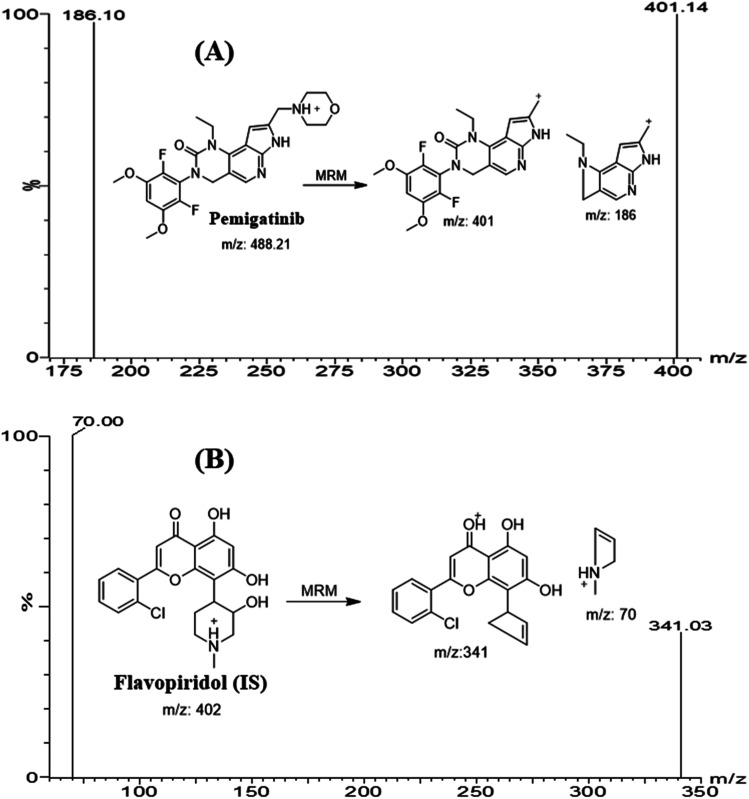
MRM mass spectra of pemigatinib (PMB) (A) and flavopiridol (internal standard; IS) (B) representing the proposed fragmentation pattern.

Mass spectrometry was conducted on triple quadrupole mass analyzer (MS/MS), and parameters were optimized to resolve PMB and flavopiridol (FLV: internal standard, IS) with good sensitivity and mass accuracy. Analysis of PMB (C_24_H_27_F_2_N_5_O_4_) and FLV (C_21_H_20_ClNO_5_) was performed in positive mode (ESI+). Tuning was performed using IntelliStart® software that were optimized manually in combined mode (fluidics and LC) to enhance peak selectivity and intensity of the studied analytes. Nitrogen (650 L h^−1^) was used as drying gas at 350 °C. The cone gas flow rate was kept at 100 L h^−1^. Argon (0.14 mL min^−1^) was utilized as a collision gas in the collision cell. Multiple reaction monitoring (MRM) mode was for quantification and to increase the sensitivity and selectivity of the established method. The cone voltage used for PMB and FLV was set as 26 (V) and 40 (V), respectively. Extractor voltage, capillary voltage, and RF lens voltage were set at 3.0 (V), 4 (kV), and 0.1 (V), respectively. The source temperature was set at and 350 °C. Dwell time for PMB and FLV mass transitions were 0.025 second. PMB peak (Rt: 1.2 min) was quantified using MRM transitions 488 → 401 (CV: 26 V and CE: 16 V) and 488 → 186 (CV: 26 V and CE: 36 V) (Fig. 1B). MRM mass transitions for FLV (Rt: 1.9 min) were 402 → 341 (CV: 40 V and CE: 24 V) and 402 → 70 (CV: 40 V and CE: 30 V) (Fig. 1A). MRM mode was utilized for detection of PMB and FLV to avoid interference from HLM matrix constituents that increased the sensitivity of the developed LC-MS/MS analytical assay.

### 2.4 Preparation of PMB working solutions

PMB and FLV (IS) showed a good solubility in dimethyl sulfoxide (DMSO) at 25 mg mL^−1^ (51.28 mM; ultrasonication and warming and heating to 60 °C), and 33.33 mg mL^−1^ (82.94 mM; ultrasonication), respectively. Stock solutions of PMB and FLV were made in DMSO at 1 mg mL^−1^ at the allowed solubility range. Stepwise dilution from the stock solutions of PMB and FLV was done using the LC-MS. PMB (1 mg mL^−1^) was diluted ten fold to prepare PMB-working solution 1 (WK1: 100 μg mL^−1^) that was diluted ten fold to prepare PMB WK2 (10 μg mL^−1^). FLV (1 mg mL^−1^) was diluted over three steps to prepare FLV WK3 (1 μg mL^−1^).

### 2.5 PMB calibration standards

DMSO was utilized because of the solubility profile of PMB in DMSO. In addition, HLM were deactivated using DMSO (2%) with slight warming (heat inactivates microsomes over 5 min at 50 °C).^22,23^ HLM were deactivated using DMSO [DMSO inhibits metabolic reactions, even at low concentrations (0.2%)^24^] before preparation of PMB standards to minimize the effect of metabolizing enzymes on PMB concentration during method validation. The HLM matrix was used at a concentration of 1 mg protein per mL by dilution of 30 μL HLM up to 1 mL with phosphate buffer (pH 7.4) containing 1 mM NADPH. PMB standards were made by diluting PMB WK2 (10 μg mL^−1^) and PMB WK3 (500 ng mL^−1^) with the HLM matrix (1 mg protein per mL) to prepare nine calibration points: 5, 15, 50, 100, 150, 200, 300, 400, and 500 ng mL^−1^ keeping the matrix volume no less than 90% of the total volume, so that the effect of dilution will be minimal. This is to ensure that the matrix is similar to the final matrix of the *in vitro* metabolic study. Four PMB levels at 5, 15, 150, and 400 ng mL^−1^ were selected as quality controls (QCs) for the validation steps: a lower limit of quantification (LLQC), a lower QC (LQC), a medium QC (MQC), a high QC (HQC), respectively. A volume of 100 μL of FLV WK3 (1 μg mL^−1^) was added as the IS to calibration standards and QCs. One hundred microliters of FLV WK3 (1 μg mL^−1^) was added as the IS to 1 mL of calibration standards and QCs.

Protein precipitation was used for extaction of PMB and FLV from the HLM matrix by adding ACN (2 mL) to the calibration standards, followed by centrifugation at 14 000 rpm for 12 min at 4 °C. The supernatant (1 mL) was filtered into 1.5 mL HPLC vials using a 0.22 μm syringe filters to ensure purity of extracts. A volume of 5 μL of filtrates was injected into the LC-MS/MS system. Control samples: HLM matrix as a blank (negative control) and HLM matrix with IS (positive control were prepared using the same procedure described above). Controls were used to verify the absence of interference from HLM matrix at the retention times of the PMB and FLV. A PMB calibration curve was constructed by plotting PMB nominal values (*x*-axis) *versus* the peak area ratio of PMB to FLV (*y*-axis). The linear regression equation (*y* = *ax* + *b*) and the coefficient of variation (*R*^2^) were used to assess linearity of the developed LC-MS/MS method.

### 2.6 Method validation

Validation parameters for the developed LC-MS/MS method were assessed following validation guidelines for bioanalytical method outlined by the USFDA general regulations and the International Conference on Harmonization (ICH). Method validation was investigated for linearity, specificity, sensitivity, accuracy, precision, matrix effect, extraction recovery, and stability.

#### 2.6.1 Specificity

Specificity of the analytical method was investigated by analyzing six blank HLM matrix samples after extraction following the proposed procedure. These samples were assayed for interferenig peaks at the elution time of PMB and FLV, and compared the chromatogram of PMB and FLV spiked HLM matrix samples. In order to minimize the effect of carryover on the mass detector, MRM mode was utilized.

#### 2.6.2 Linearity and sensitivity

Sensitivity and linearity of the developed method were evaluated using 12 calibration curves for PMB. The calibration curves were prepared on the same day of the analysis at nine concentrations in the range of 5 to 500 ng mL^−1^ in a HLM matrix. Each calibration curve was established by plotting the peak area ratio of PMB to FLV (IS) on the *y*-axis against the nominal concentration of PMB on the *x*-axis. The least squares statistical method (*y* = *ax* + *b*) was followed to generate the equation of the liner model. The LLOQ and LLOD were calculated as reported by the Pharmacopeia using the slope and the standard deviation of the intercept (SD) as follows LLOD = 3.3 × SD of intercept/slope and LLOQ = 10 × SD of intercept/slope.

#### 2.6.3 Accuracy and precision

Inter- and intra-day accuracy and precision of the developed LC-MS/MS method were investigated as per USFDA guidelines. Intraday precision and accuracy were calculated by analysis of HLM matrix samples spiked with PMB and QC samples at different levels on the first day. Interday measurements were performed in a similar way on three consecutive days. For expressing precision and accuracy of the proposed methods, percentages relative standard deviation (% RSD = SD × 100/mean) and percentages error (% error = [(average measured concentration − expected concentration)/expected concentration] × 100) were utilized for precision and accuracy respectively.

#### 2.6.4 Extraction recovery and matrix effect

Matrix effect and recovery were assessed using QC samples. The recovery of PMB from HLM was estimated by comparing peak area ratio in the mobile phase (A) and those after extraction from HLM matrix (B). The ratio of B/A × 100 is known as the % recovery. The absence of matrix effect on PMB or FLV ionization was verified by running two sets of HLM matrices. Set 1 was spiked with the PMB LQC (15 ng mL^−1^) and FLV (100 ng mL^−1^), while Set 2 was prepared by substituting the mobile phase for the HLM matrix. The matrix effects (ME) for PMB and FLV were computed using the following equation:

1 Matrix effect of PMB or FLV = mean peak area ratio Set 1/Set 2 × 100

The IS normalized ME was computed using the following equation:

2 IS normalized ME = matrix effect of PMB/matrix effect of FLV (IS)

### 2.7 PMB metabolic stability

Metabolic stability profile of PMB, including in CL_int_ and *in vitro t*_1/2_ was defined by the estimation of the remaining PMB concentration after incubation with HLM, NADPH (cofactor), and 3.3 mM MgCl_2_ in a 0.1 M sodium phosphate buffer (pH 7.4) for 50 min. Briefly, pre incubation of 1 μM of PMB was carried out using 30 μL HLM in 1 mL of 0.1 M sodium phosphate buffer (pH 7.4) at 37 °C for 10 min to set optimal a conditions for metabolic reactions. After pre incubation, NADPH (1 mM) was added to initiate metabolic reaction. To confirm results, the same metabolic experiment was repeated three times.^25^ FLV WK3 (100 μL, 1 μg mL^−1^) was added as the IS to incubation mixture just prior to the termination of the metabolic reaction in order to avoid the effect of metabolism on the IS conc. Termination of the metabolic reaction was done at specific time intervals: 0, 2.5, 7.5, 15, 20, 30, 40, and 50 min by adding 2 mL of ice cold ACN. The extraction and incubation steps were done, as stated above. Data analysis was done using the application manager ‘QuanLynx’ included with MassLynx 4.1 Software. The concentration of PMB at certain time intervals was calculated, and the PMB metabolic stability curve was determined. Considering the PMB conc. at 0 min was 100%, and the remaining PMB% was plotted against time. From this curve, linear range points were selected to establish another metabolic curve showing the natural logarithm of the percent remaining PMB over time. The slope of the linear part sl of the curve reflects the rate constant of PMB metabolism and was utilized to calculate the *in vitro t*_1/2_ using the following equation:

3 *In vitro t*_1/2_ = ln 2/slope

Next, CL_int_ (μL min^−1^ mg^−1^) was computed using the equation:^26^4
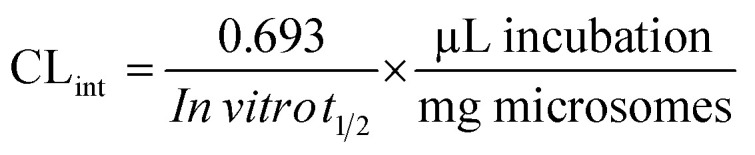


CL_int_ is then scaled to *in vivo* clearance using HLM protein concentration per gram liver and average liver weight reported in the literature.^27^

## 3 Results and discussion

### 3.1 *In silico* PMB metabolic stability

**Fig. 2:**
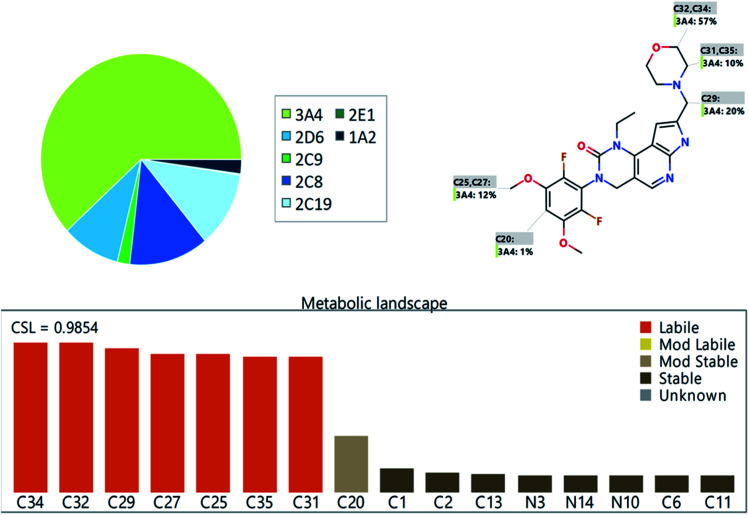
The predicted metabolic landscape of pemigatinib using StarDrop software (WhichP450 module).

The PMB metabolic landscape shows *t* metabolic lability of the active sites of PMB towards metabolism by CYP enzymes.^28–30^ This indicates that positions C29, C31, C32, C34 and C35 in the morpholine methyl group and C25 and C27 of the methoxyl group are labile to metabolism. These results and CSL (0.9854) reflect high PMB metabolism lability and therefore, the developed method was applied for PMB metabolic stability evaluation (Fig. 2).

### 3.2 LC-MS/MS method development

**Fig. 3:**
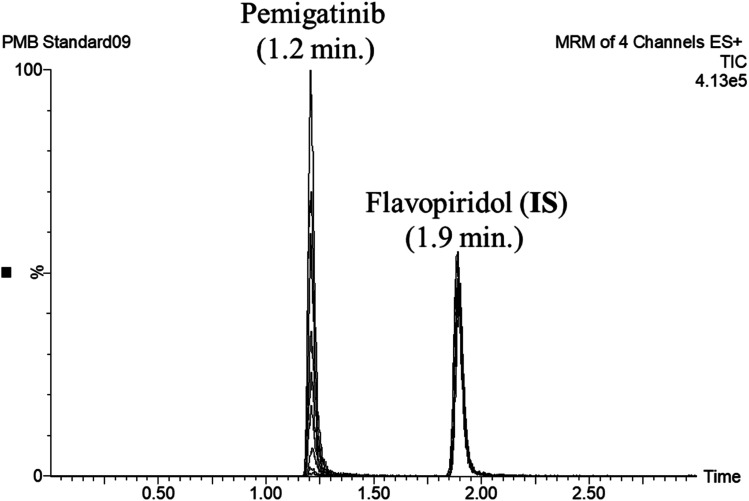
Overlaid MRM chromatograms of the PMB calibration levels showing the pemigatinib and flavopiridol (IS) chromatographic peaks at 1.2 min and 1.9 min, respectively.

FLV was chosen as the IS in PMB estimation as the protein precipitation method using ACN could be used for extracting both analytes (PMB and FLV) from the HLM matrix. PMB and FLV extraction recoveries were 101.7 ± 4.48% and 102.14 ± 3.73%, respectively. The retention times of PMB and FLV were 1.2 and 1.9 min, respectively, indicating good separation. PMB and FLV are anti-cancer drugs that would not prescribed together; therefore, the developed LC-MS/MS methodology could be used for therapeutic drug monitoring or pharmacokinetic studies of PMB, Fig. 3 shows the overlaid MRM chromatograms of PMB calibration standards.

### 3.3 Validation parameters

#### 3.3.1 Specificity

There was good resolution of analyte (PMB and FLV) peaks (Fig. 3). There was no interference from the components of HLM matrix at the analytes (PMB and FLV) elution times (Fig. 3), confirming, the specificity of the established LC-MS/MS method. In the control blank MRM chromatograms, no carry-over effect from analytes (PMB and FLV) was detected.

#### 3.3.2 Sensitivity and linearity

The established method exhibited linearity in the range of 5 to 500 ng mL^−1^ with a regression equation: *y* = 0.1471*x* − 0.3079 (weighing: 1/*x*) *R*^2^ = 0.9995. The RSD values for the six calibration curves were within <3.32% (Table 1). Back calculations for the eleven PMB standards in the HLM matrix confirmed linearity of the developed method's. The LLOQ was 5 ng mL^−1^.

**Table 1:** PMB back-calculation of six replicates of the calibration standards

PMB nominal concentrations (ng mL^−1^)	Mean^a^	SD	RSD^b^ (%)	% error
5 (LLQC)	5.41	0.17	3.10	8.27
15 (LQC)	14.75	0.49	3.32	−1.67
50	49.75	1.32	2.65	−0.51
100	103.21	1.44	1.39	3.21
150 (MQC)	150.82	1.90	1.26	0.55
200	203.92	3.62	1.78	1.96
300	298.85	3.55	1.19	−0.38
400 (HQC)	398.50	4.45	1.12	−0.37
500	502.14	3.38	0.67	0.43

a Average of six calibration curves.

b The RSD values for the six calibration curves were within <3.32%.

#### 3.3.3 Precision and accuracy

Precision and accuracy outcomes were in agreement with FDA guidelines.^31^ The intra- and inter-day accuracy and precision values of the developed LC-MS/MS method were −1.67% to 8.27% and 0.19% to 10.00%, respectively (Table 2).

**Table 2:** Intra- and inter-day (precision and accuracy) of the developed LC-MS/MS method

PMB in HLM matrix (ng mL^−1^)	Intra-day assay^a^				Inter-day assay^b^			
	5 (LLQC)	15 (LQC)	150 (MQC)	400 (HQC)	5 (LLQC)	15 (LQC)	150 (MQC)	400 (HQC)
Mean	5.41	14.75	150.82	398.50	5.50	15.34	151.33	404.75
SD	0.17	0.49	1.90	4.45	0.11	0.08	2.17	0.78
Precision (% RSD)	3.10	3.32	1.26	1.12	2.02	0.50	1.43	0.19
% error	8.27	−1.67	0.55	−0.37	10.00	2.24	0.88	1.19
Recovery (%)	108.27	98.33	100.55	99.63	110.00	102.24	100.88	101.19

a Mean of twelve repeats on the same day.

b Mean of six repeats over three days.

#### 3.3.4 PMB extraction recovery and matrix effects of HLM

The recovery of the PMB QC levels was 101.7 ± 4.48% and (RSD < 4.40%) (Table 2). The FLV recovery was 102.14 ± 3.73%. The absence of matrix effect on PMB or FLV ionization was verified by running two sets of HLM matrices. Set 1 was spiked with the PMB LQC (15 ng mL^−1^) and FLV (50 ng mL^−1^), while set 2 was prepared by substituting the mobile phase for the HLM matrix. The HLM containing PMB and FLV showed an ME of 101.28 ± 2.99% and 103.58 ± 4.23%, respectively. The IS normalized ME was 0.98 and was within the accepted range. Therefore, these results verified that the HLM matrix had no significant effect on either PMB or FLV ionization degree.

### 3.4 Metabolic stability

**Fig. 4:**
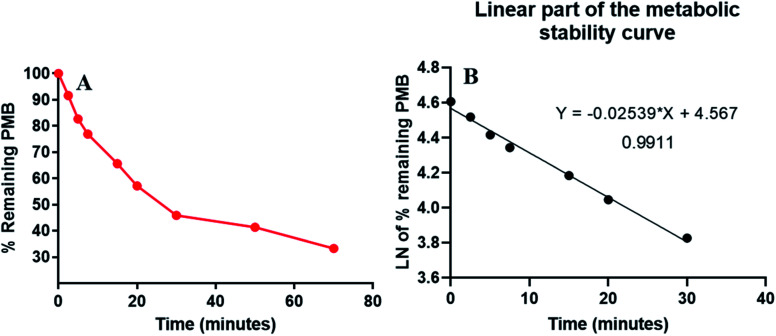
The metabolic stability curve of PMB in HLM (A) and the regression equation of the linear part of the curve (B).

PMB at 1 μM was used in incubation mixtures considered to be less than the Michaelis–Menten constant, with HLM (1 mg protein) to avoid nonspecific protein binding.^32,33^ PMB conc was computed using linear curve regression equation from a freshly prepared calibration curve. The PMB metabolic stability curve was made by plotting the incubation time (*x*-axis) against percentage PMB remaining (*y*-axis) (Fig. 4A). The linear part of the plotted curve (0–30 min) was used to construct another curve of incubation time (0–30 min) against the natural logarithm (Ln) PMB remaining (Fig. 4B). The slope of the linear portion (0.0254) described the rate constant for PMB metabolism. The linear curve regression equation was *y* = −0.0254*x* + 4.5669 with *R*^2^ = 0.9969, that was utilized for calculating PMB *in vitro t*_1/2_ (Table 3). The slope was 0.0254, so *in vitro t*_1/2_ was 27.29 min. PMB intrinsic clearance was calculated according to the *in vitro t*_1/2_ method using eqn (3), so the Cl_int_ of PMB was 25.4 μL min^−1^ mg^−1^.^34^ Based on these results, it can be proposed that PMB is a drug with a medium extraction ratio expected to have moderate accumulation in the body and potentially good bioavailability, compared with other studied tyrosine kinase inhibitors (*e.g.* dacomitinib). By utilizing the Cloe PK and simulation software, these outcomes could also be utilized to predict *in vivo* pharmacokinetics of PMB.^35^

**Table 3:** Parameters of PMB metabolic stability curve

Time (min)	Mean^a^ (ng mL^−1^)	X^b^	LN X^c^	Analytical parameters
**0**	**468.00**	**100.00**	**4.61**	Regression equation: *y* = −0.0254*x* + 4.5669
**2.5**	**428.76**	**91.61**	**4.52**	
**5**	**387.13**	**82.72**	**4.42**	*R* ^2^ = 0.9911
**7.5**	**359.86**	**76.89**	**4.34**	
**15**	**307.08**	**65.62**	**4.18**	Slope: −0.0254
**20**	**267.55**	**57.17**	**4.05**	
**30**	**214.76**	**45.89**	**3.83**	*t*_1/2_: 27.29 min
50	193.49	41.34	3.72	Cl_int_: 25.4 μL min^−1^ mg^−1^
70	155.78	33.29	3.51	

a Average of three repeats.

b X: average of the percentage PMB remaining from three repeats.

c The linear range is indicated by bold font.

## 4 Conclusions

A validated LC-MS/MS method was developed to assess PMB metabolic stability. The LC-MS/MS method exhibited good sensitivity, eco-friendliness (due to using less organic solvent), high recovery, accuracy, and rapid analysis. Our findings revealed that PMB an *in vitro t*_1/2_ values of 27.29 min and a moderate CL_int_ (25.4 μL min^−1^ mg^−1^), which suggest a moderate rate of hepatic clearance. Accordingly, an acceptable *in vivo* bioavailability could be predicted. From these outcomes, we propose that PMB could be given to patients without the effect of rapid excretion through the liver or dose accumulation inside the human blood. The *in vitro* metabolic experiments data was used to confirm the outcomes of the *in silico* predictions. Future studies may be required to test this approach for *in vivo* therapeutic drug monitoring.

## Ethical statement

The study design using *in vitro* experiments with commercially available human liver microsomes exempts it from the need of the Ethics Committees approval.

## Author contributions

All authors did considerable contributions to the design and conception, data acquisition, or analysis and data interpretation; participated in article drafting and revising critically for significant intellectual content; approved submission to the current journal; gave final approval of the published version; and agreed to be responsible for all parts of the work.

## Abbreviations

PMB: Pemigatinib
FLV: Flavopiridol
IS: Internal standard
ESI: Electrospray ionization
HLM: Human liver microsomes
LC-MS/MS: Liquid chromatography tandem mass spectrometry
EGFR: Epidermal growth factor receptor
*t*_1/2_
: Half-life

## Conflicts of interest

The authors report no conflicts of interest for this work.

## Supplementary Material
